# Physical activity, sugar-sweetened beverages, whole grain bread and insomnia among adolescents and psychological distress in adulthood: prospective data from the population-based HUNT study

**DOI:** 10.1186/s12966-021-01215-7

**Published:** 2021-11-01

**Authors:** Annette Løvheim Kleppang, Karin de Ridder, Siri Håvås Haugland, Tonje Holte Stea

**Affiliations:** 1 Department of Health and Nursing Science, University of Agder, Postboks 422 4604, Kristiansand, Norway; 2 Department of Epidemiology and public health, Sciensano, Brussels, Belgium; 3 Department of Psychosocial Health, University of Agder, Kristiansand, Norway; 4 Department of Child and Adolescence Mental Health, Sørlandet Hospital, Kristiansand, Norway

**Keywords:** Lifestyle behaviours, Physical activity, Sugar-sweetened beverages, Whole grain bread, Insomnia, Psychological distress, Prospective data, The HUNT study

## Abstract

**Background:**

In this study, we examined the relationship between low levels of physical activity, high consumption of sugar-sweetened beverages and low consumption of whole grain bread and experiencing insomnia in adolescence and psychological distress in young adults.

**Methods:**

This prospective study was based on information retrieved from the Trøndelag Health Study (HUNT) in Norway and included adolescents (age 13–19) participating in Young-HUNT3 (2006–2008) and in HUNT4 (2017–2019) 11 years later (age 23–31). The study sample consisted of 2,230 participants (1,287 females and 943 males). The exposure variables collected in adolescence included self-reported physical activity, consumption of sugar-sweetened beverages and whole grain bread and insomnia, and psychological distress in young adulthood was used as an outcome variable. The relationship between lifestyle behaviours in adolescence and psychological distress in young adulthood was examined using multivariable logistic regression, adjusted for gender, age and psychological distress in adolescence and educational level in young adulthood.

**Results:**

An increased odds of psychological distress was shown among young adults who reported low levels of physical activity (OR: 1.44, 95 % CI: 1.10–2.89), high consumption of sugar-sweetened beverages (OR: 1.49, 95 % CI: 1.12–1.98), low consumption of whole grain bread (OR: 1.35, 95 % CI: 1.04–1.77) and insomnia (OR: 1.69, 95 % CI: 1.23–2.33) in adolescence. In terms of absolute differences, unhealthy lifestyle behaviours increased the risk of psychological distress in young adulthood between 3.18 (95 % CI: 0.29–6.07) (low whole grain bread consumption) and 6.01 (95 % CI: 1.95–10.07) (insomnia) percentage points.

**Conclusions:**

Low levels of physical activity, high consumption of sugar-sweetened beverages and low consumption of whole grain bread and insomnia during adolescence were associated with psychological distress in young adulthood.

## Background

The prevalence of mental disorders has been associated with increased rates of mortality [1] and disability-adjusted life years [2]. Common mental disorders, such as depressive disorders and anxiety disorders, are highly prevalent among young adults in their 20 s, and those suffering from a mental disorder in their 20 s have an increased risk of experiencing a mental disorder later in life [3]. The first onset of mental health disorders appears to start in childhood or adolescence and may persist throughout life [4]. Thus, from a public health perspective, it is crucial to identify any modifiable factors preceding the development of mental health problems in adulthood.

Healthy lifestyle behaviours have been associated with improved psychological well-being, lower psychological distress and fewer mental health difficulties [5, 6], and the importance of focusing on lifestyle factors in the prevention and treatment of mental illness have been highlighted in a recent meta-review [7]. Moreover, a recent population-based prospective study among Canadian children and adolescents confirmed that adherence to lifestyle recommendations developed to promote physical health in children and prevent chronic diseases later in life also had short-term benefits for mental health and may reduce the future burden of mental illness [8]. A study among French adults also showed that increasing the number of healthy lifestyle behaviours was associated with a lower risk of depressive symptoms [9]. However, most previous studies examining the association between lifestyle behaviours and psychological distress have been cross-sectional, and few studies have examined the longitudinal relationship between adolescence and young adulthood.

Lifestyle behaviours include a broad range of behaviours, and to identify the potentially potent areas for preventing psychological distress, it may be useful to focus on the everyday lifestyle practices and choices that most adolescents face, such as physical activity, dietary patterns and sleep. The results from a longitudinal study and meta-analysis of prospective studies indicated that physical activity may have a preventive effect on the development of depression [10, 11]. Another prospective cohort study reported that low levels of physical activity in adolescents were associated with poor mental health in early adulthood among females but not for males [12]. On the other hand, the results from other prospective studies did not find longitudinal associations between physical activity and the symptoms of depression and anxiety [13, 14], while other prospective studies reported an inverse association between sport participation and symptoms of depression and anxiety [15, 16]. The inconsistency in these findings calls for further longitudinal studies.

Evidence has also confirmed the importance of the relationship between dietary patterns or quality and mental health early in the lifespan [17]. Several prospective studies and a recent meta-analysis concluded that the consumption of soft drinks is a major risk factor for developing depression among adults [18–21], and similar findings were shown among Norwegian adolescents [22]. Another longitudinal study showed that reducing adolescents’ intake of soft drinks resulted in reduced aggressive behaviour but no changes in depressive symptoms [23]. No significant association between sugar-sweetened beverage consumption and depression risk was found in a prospective cohort study (10-year follow-up) among Spanish students, but the results indicated that the consumption of sugar and overall low carbohydrate quality was associated with an increased risk of depression [21]. A recent review has also reported that diets rich in omega-3-polyunsaturated fatty acids and dietary fibre may reduce the risk of developing the symptoms of depression and anxiety [24]. A cross-sectional study among adults revealed an inverse association between the consumption of whole grains and depression scores [25]. To the best of our knowledge, it remains unknown what the unique preventive potential of dietary fibre and soft drink consumption during adolescence might be for reducing the risk of psychological distress later in young adulthood.

In addition, several studies have indicated that total sleep time and sleep quality in adolescence seem to predict psychological distress [26, 27], depression and anxiety [28–30] and that sleep problems frequently co-occur with anxiety and depression [26]. Moreover, a recent longitudinal study among youth showed that adherence to sleep recommendations was the most consistent predictor of depressive symptoms [31]. However, a recent cross-sectional study among 60 adolescents found no significant relationship between sleep duration and psychological distress [27]. Because of inconclusive findings, a recent study emphasised the importance of future research examining the relationship between sleep habits in adolescence and anxiety and depression later in life [26].

Both low socioeconomic status (SES) and mental health difficulties in childhood have also been associated with poor lifestyle beahviour [32, 33], as well as mental health problems [33–35]; therefore, mental health difficulties in adolescence and SES should be taken into account when examining the relationship between different lifestyle behaviours and mental health.

The evidence for which lifestyle behaviours in adolescence should be addressed when aiming to prevent the onset of psychological distress is currently limited and inconsistent. To the best of our knowledge, few studies have assessed the relative influence of multiple modifiable lifestyle behaviours in adolescence on psychological distress in young adulthood. Thus, the purpose of the current study was to examine the relationship between low levels of physical activity, high consumption of sugar-sweetened beverages and low consumption of whole grain bread and insomnia in adolescence and psychological distress in young adulthood.

## Methods

The present prospective study was a part of the Trøndelag Health Study (HUNT). Data collection for the first measurements, which covered the adolescent part, was conducted in 2006–2008 (Young-HUNT3), whereas the second wave of the HUNT study, which covered the adult part, was conducted in 2017–2019 (HUNT4). All adolescents aged 13–19 years living in the county of Trøndelag in Norway were invited to participate in the Young-HUNT3 study by completing a self-reported questionnaire during school hours. In 2017–2019, 11 years later, the same population (now aged 23–31) were invited to participate in a fourth wave of the adult part of the HUNT study (HUNT4) and completed this survey by filling out a comprehensive health-related questionnaire at home. Participation was voluntary, and the respondents were informed that they could withdraw from the studies at any time. The HUNT study is a collaboration between the HUNT Research Centre (Faculty of Medicine and Health Sciences, Norwegian University of Science and Technology NTNU), Trøndelag County Council, Central Norway Regional Health Authority and the Norwegian Institute of Public Health.

**Fig. 1:**
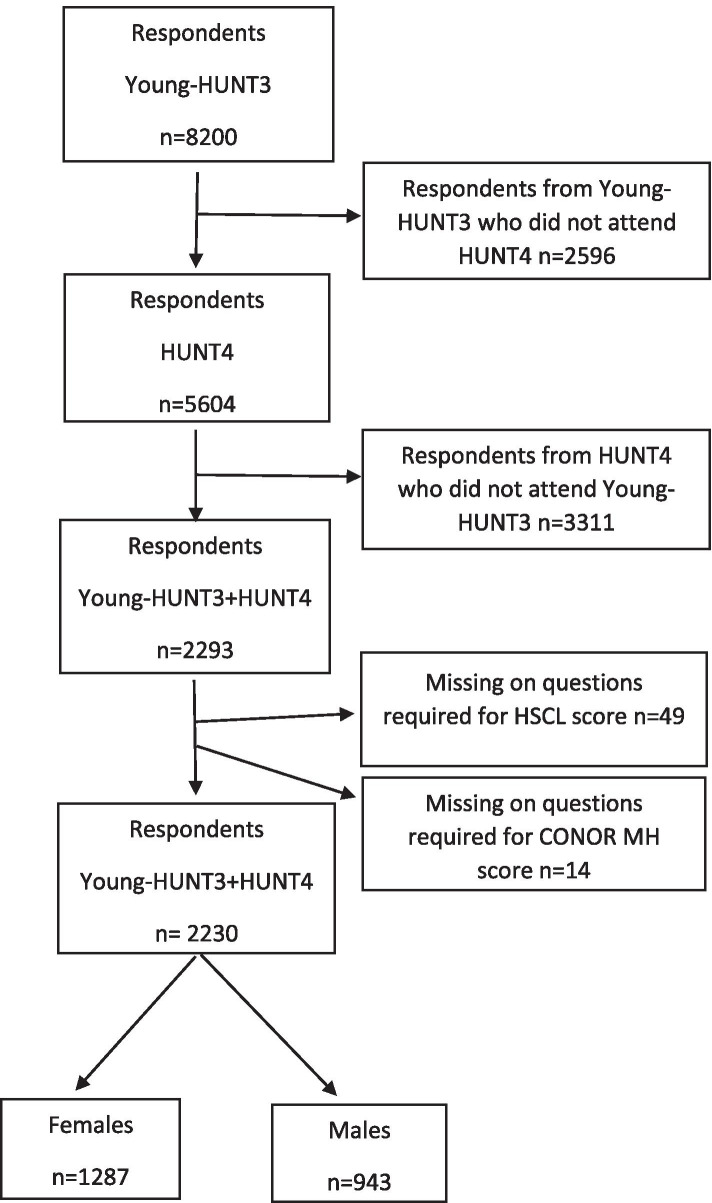
Flowchart for the study population: Norwegian HUNT studies, Young-HUNT3 in 2006–2008 and HUNT4 in 2017–2019

In the Young-HUNT3 study, 8,200 respondents agreed to participate (78.4 % of all invited) and in HUNT4, 56,078 respondents agreed to participate (54 % of all invited). In the present study, those participating in both Young-HUNT3 and HUNT4 were included, resulting in a total of 2,293 respondents (1,320 females and 973 males). Finally, after excluding those with missing information on any HSCL-5 items or CONOR MH items, the current study comprised 2,230 respondents(1,287 females and 943 males). The flowchart in Fig. 1 shows the population in 2004–2006 and 2017–2019.

### Measures

### Outcome variable—young adult psychological distress

In HUNT4, psychological distress was assessed by the Conor Mental Health Index (CONOR-MHI). This measurement consists of seven items reflecting the various aspects of psychological distress. The CONOR-MHI is a modified version of the General Health Questionnaire [36] and Hopkin’s Symptoms Checklist [37]. The respondents were asked if during the last two weeks they had felt ‘nervous and unsettled’, ‘troubled by anxiety’, ‘secure and calm’, ‘irritable’, ‘happy and optimistic’, ‘sad/depressed’ or ‘lonely’. Each question had four answer categories, ranging from ‘No’ (1) to ‘Very’ (4). The average CONOR-MHI score was calculated by dividing the total score by seven (number of items). Missing values were replaced with the sample mean value for each item. Records with two or more missing items were, however, excluded (see Fig. 1). In the present study, a cut-off point of ≥2.15 was used to determine psychological distress, which is considered a valid cut-off value for the prediction of psychological distress [38].

### Exposure variables—lifestyle behaviours in adolescence

The respondents were asked the following: ‘Outside of school hours, how many hours do you usually exercise in your free time so much that you get out of breath or sweat?’ The response options were as follows: ‘Never’, ‘Approximately 30 min a week ‘Approximately 1–1 ½ hours a week’, ‘Approximately 2–3 hours a week’, ‘Approximately 4–6 hours a week’ or ‘7 hours and more per week’. A high level of physical activity was defined as ‘Four hours or more per week’ (reference category), and ‘Less than 4 hours’ was defined as a low level of physical activity.

The consumption of sugar-sweetened beverages was measured by the following: ‘How often do you drink the items listed below?’ The response alternatives were ‘Seldom/never’, ‘1–6 glasses a week’, ‘1 glass a day’, ‘2–3 glasses a day’ and ‘4 or more glasses a day’. Consumption of whole grain bread was measured by the following question: ‘How often do you eat the items listed below?’ The response alternatives were ‘Several times a day’, ‘Once a day’, ‘Every week but not every day’, ‘Less than once a week’ and ‘Never’. Consumption was dichotomised into ‘Daily consumption and more’ (reference category for whole grain) and ‘Less than daily consumption’ (reference category for sugar-sweetened soft drinks).

Classification of insomnia was based on two questions, as follows: ‘Have you had problems falling asleep during the last month?’ and ‘During the last month, did you ever wake up too early, not being able to fall asleep again?’ The following response options were given: ‘Almost every night’, ‘Often’, ‘Occasionally’ and ‘Never’. The respondents were classified with insomnia if they answered ‘Often’ or ‘Almost every night’ on at least one of the questions and with no insomnia if they answered ‘Less than often’ (reference category) for both questions, which is in accordance with former research [39, 40].

### Control variables

#### Age of the adolescents

The age of the respondents in YoungHUNT3 varied between 12.8 and 20.9 years and was dichotomised as ≤ 16 years and >16 years (reference category).

### Highest education as young adults

Education was assessed by the following question (HUNT4): ‘What is your highest level of education?’ and divided into four categories: ‘Primary school’, ‘High school’, ‘College ≤4 years’ and ‘College >4 years’. Educational level as young adults was dichotomised into ‘No higher education’, including primary school and high school) and ‘Higher education’, including college ≤4 years and college >4 years (reference category).

### Psychological distress in adolescence

In the YongHUNT3, psychological distress was assessed by the Hopkins Symptom Checklist-5 (HSCL-5), a five-item shortened version of the HSCL-25. HSCL-25 is a widely applied screening tool designed to measure the symptoms of depression and anxiety [41]. The HSCL-5 has been shown as a reliable and valid short form of the HSCL-25 and has been recommended as a screening instrument for symptoms of depression and anxiety [42, 43]. The respondents were asked if during the last 14 days they had been affected by the following: ‘constantly afraid and anxious; felt tense or uneasy; felt hopeless about the future; felt dejected or sad; worried too much about various things’. The five questions had four response alternatives, ranging from ‘Not bothered’ (1) to ‘Very much bothered’ (4). The responses were summarised across all items, and the mean score was used as a measure of psychological distress. In the present study, a cut-off point at >2.0 was used to determine psychological distress, which is considered a valid cut-off value for the prediction of psychological distress in adolescents [43]. We excluded the respondents with missing values on at least one of the HSCL-5 items (Fig. 1).

### Analyses

Analyses were carried out using SPSS 25.0 and STATA 16.0 for Windows. Baseline characteristics and lifestyle behaviours stratified by gender were presented as proportions with a 95 % confidence interval (CI) in each stratum.

Binominal logistic regression analyses were used to explore the association between low levels of physical activity, high consumption of sugar-sweetened beverages and low consumption of whole grain bread and experiencing insomnia during adolescent and psychological distress in young adults. All four models were adjusted for gender, age, psychological distress in adolescence and highest education in young adulthood. The associations were presented as odds ratios (OR) with a 95 % CI. Furthermore, logistic regression models were used to estimate the absolute risk differences at the follow-up 11 years later with the covariates as their mean. A risk difference describes how a 1-unit change in an independent variable (e.g., physical activity or not) alters the absolute risk of a current outcome (psychological distress). The risk difference focuses on the absolute effect of the risk factor (e.g., low level of physical activity) on the excess risk of disease (e.g., psychological distress) in those who have a risk factor compared with those who do not. From a public health point of view, this information might be more informative than a relative estimate like the OR, which examines the strength of an association.

An interaction analysis was used to examine the influence of gender on the strength of the relationship between physical activity and psychological distress. The possible interaction effects were examined using LR tests (likelihood ratio test) by contrasting models with and without interaction terms. The main effect model included physical activity, gender age, psychological distress and highest education as the independent variables and was tested against models, here also including interactions between physical activity by gender. A parallel interaction analysis was carried out for sugar-sweetened beverages, whole grain bread and insomnia. The incremental change in the log likelihood between the main effect models and the four models, including interactions, was not significant. Thus, the fit was not improved with the other interaction models. Therefore, only the main effect model has been presented in the results.

## Results

Table 1 presents the differences in psychological distress between males and females in young adulthood with unhealthy lifestyle behaviours during adolescence and background characteristics.

**Table 1:** Psychological distress in young adulthood among males and females with unhealthy lifestyle behaviors during adolescence and background charactertics

Variables	Males (*n* = 943)	Females (*n* = 1287)
	High level of psychological distress (*n* = 90 ) % (95% CI)	High level of psychological distress (*n* = 182) % (95% CI)
Young-HUNT3 2006-2008		
**Lifestylehabits**		
***Physical activity***		
< 4 hours per week after school	10.2 (7.3-13.0)	17.7 (14.9-20.6)
***Sugar-sweetened beverages***		
Daily consumption	11.3 (8.1-14.4)	19.2 (14.4-23.9)
***Whole grain bread***		
Less than daily consumption	12.4 (8.7-16.2)	17.5 (13.9-21.0)
***Sleeping***		
Insomnia	8.0 (6.1-10.0)	12.4 (10.3-14.4)
***Psychological distress (HSCL-5)***		
High level (>2.0)	22.0 (11.5-32.6)	25.2 (19.7-30.7)
***Age***		
≤ 16	12.2 (9.5-15.0)	15.3 (12.6-17.9)
HUNT4 2017-2019		
***Education level as young adults***		
No higher education	11.2 (8.7-13.7)	18.1 (14.8-21.5)

The dependent variable consistent of two categories coded as low level of psychological distress (CONOR MHI < 2.15) in young adults (HUNT4 2017-2019) and high level of psychological distress (CONOR MHI ≥2.15)

The results in Table 1 show that among adolescents with a low level of physical activity, high consumption of sugar-sweetened beverages, low consumption of wholegrain bread and insomnia, a higher proportion of females than males reported a high level of psychological distress in young adulthood (17.7 % vs. 10.2 %, 19.2 % vs. 11.3 %, 17.5 % vs. 12.4 % and 12.4 % vs. 8.0 %, respectively). Similarly, among adolescents with high level of psychological distress, a higher proportion of females than males reported a high level of psychological distress in young adulthood (25.2 % vs. 22.0 %, respectively). Further, among young adults with a low level of higher education, a higher proportion of females than males reported psychological distress (18.1 % vs. 11.2 %, respectively).

In Table 2, the regression analysis for each lifestyle behaviour in adolescence shows the OR and risk differences of psychological distress in adulthood.

**Table 2:** Odds ratio and absolute risk differences^a^ of psychological distress in adulthood in relation to lifestyle behaviours in adolescence

	Crude model^**a**^	Adjusted model^**a**^	Crude model	Adjusted model
Variables	OR	OR	Risk differences^**a**^	Risk differences
***Physical activity***				
≥ 4 hours per week after school	1 (ref)	1 (ref)		
< 4 hours per week after school	1.63 (1.25–2.11)	1.44 (1.10–1.89)	5.09 (2.41–7.77)	3.68 (0.95–6.42)
***Sugar-sweetened beverages***				
Less than daily consumption	1 (ref)	1 (ref)		
Daily consumption	1.34 (1.03–1.75)	1.49 (1.12–1.98)	3.25 (0.16–6.35)	4.30 (1.07–7.52)
***Whole grain bread***				
Daily consumption	1 (ref)	1 (ref)		
Less than daily consumption	1.57 (1.21–2.04)	1.35 (1.04–1.77)	5.00 (1.98–8.02)	3.18 (0.29–6.07)
***Insomnia***				
No insomnia	1 (ref)	1 (ref)		
Insomnia	2.30 (1.72–3.08)	1.69 (1.23–2.33)	10.62 (6.25–14.99)	6.01 (1.95–10.07)

Crude: Bivariate analysis. OR: odds ratio; 95% CI. Adjusted; Adjusted for gender, age, psychological distress in adolescence and highest education as young adults from the main effects model. ^a^Estimated risk differences (percentage points, with 95% CI) for psychological distress for each lifestyle behavior with the covariates as their mean

A higher odds of psychological distress was observed among those with a low level of physical activity (OR: 1.44, 95 % CI: 1.10–1.89), high consumption of sugar-sweetened beverages (OR: 1.49, 95 % CI: 1.12–1.98), low consumption of whole grain bread (OR: 1.35, 95 % CI: 1.04–1.77) and insomnia (OR: 1.69, 95 % CI: 1.23–2.33) compared with those with more healthy physical activity, dietary and sleeping behaviours. In terms of absolute differences, unhealthy lifestyle habits increased the risk of psychological distress in young adulthood between 3.18 (95 % CI: 0.29–6.07) (less whole grain bread) and 6.01 (95 % CI: 1.95–10.07) (insomnia) percentage points.

**Fig. 2:**
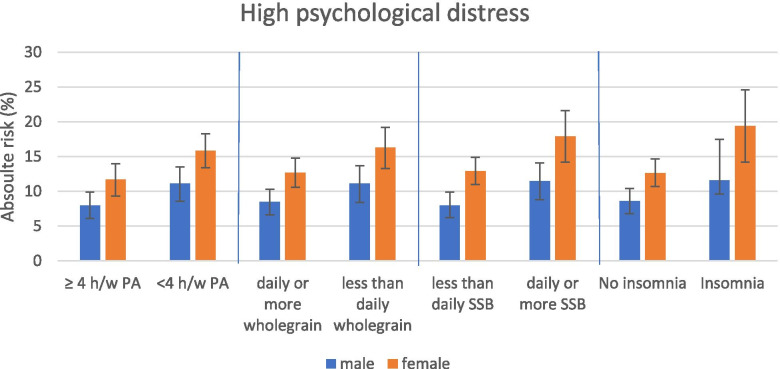
Estimated 11-year risk of psychological distress among young adults according to lifestyle behaviours in adolescence and gender. SSB: sugar-sweetened beverages

The increase in the risk differences of psychological distress according to the different lifestyle behaviours and genders are shown in Fig. 2. For both genders, the risk differences for the development of psychological distress in young adulthood was statistically significant for all examined lifestyle behaviours.

## Discussion

Our main findings indicate that unhealthy lifestyle behaviours, including low levels of physical activity, high consumption of sugar-sweetened beverages, low consumption of whole grain bread and insomnia in adolescence, increase the risk of psychological distress in young adulthood by 3 to 6 % points. Our results may not be directly comparable to the results from previous studies because of differences in population, study design and measures of lifestyle behaviours and psychological distress. However, some similarities and differences can be seen. A prospective study among adolescents found that adherence to lifestyle recommendations for physical activity, sedentary behaviour, diet and sleep had short-term benefits for mental health problems [8].

In general, women reported more psychological distress in young adulthood than men. Additionally, for all examined lifestyle behaviours, we observed an additive effect of unhealthy lifestyle for both genders. A low level of physical activity in adolescence is associated with an increased risk of psychological distress in young adulthood. The results from another study showed that a low level of physical activity in adolescence (14 and 15 years of age) was associated with poor mental health seven years later in females but not males [12]. Another study among Dutch adolescents did not find evidence indicating that physical activity may have protective effects on depressive symptoms, regardless of gender [44]. The inconsistency in these findings may be because of variations in the follow-up period and differences in age at the follow-up.

The current study showed that the high consumption of sugar-sweetened soft drinks in adolescence was associated with more psychological distress for both genders in adulthood. This is in line with prospective studies showing that the high consumption of sugar-sweetened beverages is related to mental health problems in adolescence [45] and depressive symptoms in adulthood [19, 21, 46]. However, the present study revealed that on average, women with daily or high consumption of sugar-sweetened beverages have a 5.0 % points higher risk of psychological distress in young adulthood than men. A study among children and adolescents did not find longitudinal effects of soft drink consumption on mental health problems but revealed that the prevalence of mental health problems predicted soft drink consumption over an average of six years [47]. Additionally, higher exposure to added sugars and poor quality of carbohydrates (measured by an overall carbohydrate quality index) were associated with a higher risk of depression in a prospective cohort study; however, no significant association between the consumption of sugar-sweetened beverages and depression risk was found [21]. High consumption of soft drinks has been suggested as a dysfunctional method of emotion-focused coping with mental health problems [47]. A possible biological mechanism for the association between sugar-sweetened beverages and psychological distress might be related to the chronic systemic inflammation induced by sugar [48, 49]. The mechanisms by which a proinflammatory diet could increase the risk of psychological distress may be through proinflammatory nutrients activating the innate immune system, which can lead to low-grade inflammation and mental health disorders [50].

The present study also showed that the high consumption of whole grain bread in adolescence was associated with decreased risk of psychological distress in young adulthood for both genders. To the best of our knowledge, few longitudinal studies have examined the relationship between whole grain bread consumption in adolescence and psychological distress in adulthood. In the Young-HUNT cohort, low whole grain bread consumption in adolescence was associated with a high risk of psychological distress 11 years later. A longitudinal study among adults found that the high consumption of nonrefined grain was significantly related to lower depression and anxiety [51]. Also, for whole grain bread consumption, we observed an additive effect of gender and unhealthy behaviour. The gender-stratified analyses in the present study also revealed that females with low consumption of whole grain bread had a 3.6 % points higher risk for psychological distress than men. Similarly, another prospective study among women found that the consumption of whole grain foods was inversely associated with depression in women [52]. Whole grain bread is an important source of nutrients, together contributing with other whole grain products to approximately 50 % of the fibre intake in the Norwegian population [53]. A recent meta-analysis found that increased intake of dietary fibre was associated with a lower risk of depression in adults [54]. Similarly, a cross-sectional study among Spanish children showed an association between the increased intake of fibre and lower levels of depressive symptoms [55]. The mechanism linking psychological distress to dietary fibre is unclear, but a recent review suggested that dietary fibre lowers inflammation by modifying both the permeability of the gut and pH level, and a reduction in inflammatory compounds may alter neurotransmitter concentration to reduce depressive symptoms [56]. Additionally, whole grain bread contains fatty acids, which have been linked to an increased risk of depressive symptoms, but they also contain zink, folic acid and B6, which may be inversely associated with depression have been shown to have a protective effect against and mental health symptoms in different populations [57, 58].

Finally, the present study revealed an association between insomnia and a higher prevalence of psychological distress for both genders, which is in line with previous studies [27, 29]. Moreover, one study by F. Orchard, et al. [26] confirmed that sleep quality and total sleep time at age 15 predicted anxiety and depression symptoms (and diagnoses) at ages 17, 21 and 24. Prospective studies and a review of prospective studies have also shown that not getting an adequate amount of sleep was associated with symptoms of depression, DSM-IV major depression [28, 31, 59] and anxiety symptoms [30]. Sleep problems frequently co-occur with anxiety and depression and are common in adolescents [26]. A prospective study examining the association between sleep deprivation and symptoms of depression and major depression found reciprocal effects for major depression and sleep deprivation but not for depressive symptoms [59]. Short sleep duration and insomnia seem to be not simply symptoms of physical or mental illness, but also more predictive of chronic mental health symptoms [60]. Gender differences were also identified in the present study: females with insomnia problems had a 6.7 % points higher risk for psychological distress than men with insomnia problems. Studies examining the mechanisms linking sleep and psychological distress are scarce. A review by Blake et al. [61] concluded that biological, psychological and social mechanisms underlie insomnia and internalising symptoms (anxiety and depression) in adolescence.

### Strengths and limitations

A strength of the current study is the longitudinal design, which is based on a large, representative population of Norwegian adolescents. The long follow-up period (11 years) made it possible to prospectively examine the association between physical activity, consumption of whole grain bread and sugar-sweetened beverages and insomnia in adolescence and psychological distress in young adulthood. This is a strength compared with previous studies, which have generally been restricted to the adolescent or adult period and have short follow-up periods.

Furthermore, the analyses were adjusted for well-known confounders such as gender, age, psychological distress in adolescence and a higher educational level in adulthood. However, we cannot exclude the possible residual confounders attributable to unknown or unmeasured factors; for example, when assessing the longitudinal associations of diet and beverage consumption during a week in adolescence, diet may also be a confounder. The measures are based solely on self-reports, which can be prone to recall bias, especially concerning items measuring lifestyle behaviours. For example, participants tend to over-report physical activity in self-report measures when compared with objectively measured activity [62]. There is the potential for selection bias because the participants must have attended both HUNTH3 and HUNT4 to be included. Previous analyses have shown that those not participating in HUNT studies have lower education and worse health [63]. A comparison of educational level between the subpopulation included in our study with the total HUNT4 population revealed a further selection of individuals with higher educational levels (50 % vs., 39 %, respectively) [64]. Information on physical activity, consumption of whole grain bread and sugar-sweetened beverages and insomnia was not updated throughout the follow-up period. Additionally, the interpretation of whole grain bread by the participants may not always be correct because of the use of malt and sirup (‘colouring’) to provide more moisture and increase the shelf life of the bread. A brown bread can be defined by the participants as ‘whole grain bread’, but in reality, it may not be.

## Conclusions

Insomnia at ages 13–19 seems to predict psychological distress 11 years later. The same patterns were observed for low physical activity, low consumption of whole grain bread and high consumption of sugar-sweetened beverages. On average, the risk of psychological distress is increased for both genders in the case of unhealthy behaviour. This knowledge is important to develop generic prevention approaches to reduce mental health problems and strengthen mental health in adolescence and in adults.

## Data Availability

The HUNT Research Centre has permission from the Norwegian Data Inspectorate to store and handle these data. To protect the participants’ privacy, the HUNT Research Centre aimed to limit storage of data outside the HUNT databank and cannot deposit data in open repositories. The HUNT databank has precise information on all data exported to different projects and can reproduce these on request. There are no restrictions regarding data export given approval of applications to HUNT Research Centre. For more information, see http://www.ntnu.edu/hunt/data.

## Abbreviations

HSCL-5: Hopkins Symptoms Checklist-5
CONOR-MHI: Conor mental health index
OR: Odds ratio
HUNT: Trøndelag Health Study
CI: Confidence interval
SES: Socioeconomic status
SSB: Sugar-sweetened beverages
